# Frontal theta power prospectively associated with response inhibition

**DOI:** 10.1162/IMAG.a.1390

**Published:** 2026-10-13

**Authors:** Daan van Rooij, Sam van Bijnen, Iris Schutte, Nathan van der Stoep, J. Leon Kenemans

**Affiliations:** Experimental Psychology, Helmholtz Institute, Utrecht University, Utrecht, Netherlands; Human Machine Teaming, Defense, Safety, and Security, TNO, The Hague, Netherlands

**Keywords:** response inhibition, proactive inhibition, stop-signal performance, frontal theta power

## Abstract

A proactive mechanism has been postulated to promote successful inhibition. Specifically, this mechanism is thought to operate before any action-demanding or countermanding event has occurred. In the current study, we investigated whether theta-range oscillatory power measured using electroencephalography (EEG) could reflect this mechanism in a sample of healthy individuals performing a stop-signal task. We tested whether frontal-midline (FM) theta power preceding failed versus successful stop trials predicts inhibition success. Results from two experiments demonstrate that FM theta is indeed higher preceding successful stopping events than failed ones. However, theta power turned out to be equally stronger preceding slow than fast, go trials, suggesting that proactive slowing could explain the association between theta and stop success. There was no correlation between the strength of the within-subject theta–stop-success association and the within-subject theta-RT association. In sum, group-average-level analysis suggests strong overlap between FM theta associated with proactive inhibition and proactive slowing, respectively, while at the same time there are dissociations in terms of individual differences. A further, unexpected, finding was that FM alpha power was specifically associated with proactive inhibition but not with slowing, and, relative to theta, peaked closer in time to the action-demanding event.

## 1 Introduction

Response inhibition is an important mechanism for suppressing a prepared or prepotent response tendency. Previously, we postulated that response inhibition involves two components: a proactive cognitive-control component and a reactive component (Kenemans, 2015). The reactive component refers to the ability to process and react to an unexpected event, whilst the proactive component serves to prepare sensory-specific neural pathways that facilitate response suppression, should this prove necessary (Kenemans, 2015; see also Cai et al., 2011; Majid et al., 2013). Response inhibition is preferably assessed using a stop-signal task (SST; Verbruggen et al., 2019), in which participants respond to various go stimuli, which are occasionally followed within a split second by a stop signal, signaling the participants to suppress the ongoing response tendency. From the stop-signal task, a stop-signal reaction time (SSRT) can be derived, which reflects the time needed to process the stop signal and suppress the response that was prepared.

Theta oscillations derived from EEG over frontal cortical regions (frontal-midline or FM theta; Messel et al., 2021) are associated with cognitive control (Cavanagh & Frank, 2014; Zavala et al., 2018). However, it is unclear whether FM theta also reflects cognitive control in the context of response inhibition. Several previous findings indicate that theta power is indeed associated with proactive cognitive control. For example, increased theta power has been shown to be elicited by cues that inform about an upcoming event (as compared with non-informative cues; Mazaheri et al., 2010) or the most likely response to an event (Mazaheri et al., 2014), as well as by cues that signal highly probable subsequent response conflict (van Driel et al., 2015). The same holds for cues that signal task switching. Such cues elicit stronger theta connectivity (Cooper et al., 2015), or theta power (Cooper et al., 2017, 2019), relative to repeat-task cues. In the context of inhibition, one study did observe differences in pre-trial theta as a function of no-go-probability in a go/no-go task, showing higher theta preceding trials with a low no-go probability (Adelhöfer & Beste, 2020). However, as noted by Aron (2011), in a go/no-go paradigm, one cannot be sure when the Go and Stop processes begin. As a result, the timing of any proactive response-inhibition mechanism becomes uncertain.

If a proactive component does exist for response inhibition in a stop-signal task, it could manifest as enhanced frontal theta power immediately preceding go stimuli in a stop-signal context. Theta power would then reflect the process of cognitive preparation for a potential inhibition event. Following Kenemans (2015): The reactive component of response inhibition would be implemented as a frontal (f) P3 stop effect (i.e., the difference in fP3 to the stop signal between successful and failed stops), most likely implemented in the pre-supplementary motor area (preSMA). The proactive component should also be visible from an association with stopping success, but now preceding in time each go-stop configuration. This would reflect proactive preparation in prefrontal regions, potentially including the right inferior frontal gyrus (Aron & Poldrack, 2006). Such a specific contribution from right-frontal regions would be consistent with the results from Asanowicz et al. (2022), where a separate right-hemisphere theta source was observed. Hence, we expected to observe increased theta power measured above prefrontal areas before trials preceding successful as compared with failed stops. To the best of our knowledge, the association between frontal theta and stopping performance has not been investigated in an SST paradigm.

It should be noted that it is important to distinguish proactive inhibition from proactive slowing. It has been reliably demonstrated that in SST paradigms, participants often show proactive slowing of their reaction to the go-stimulus in anticipation of an expected stop event (Pas et al., 2017, 2021; Vink et al., 2014; Zandbelt et al., 2013). This can be observed as longer reaction times with higher stop-signal probability. However, an increase in proactive inhibitory control would be associated with faster inhibition and hence shorter SSRT but not with longer reaction times on go-trials (go-RT). This would constitute a yet further instance of dissociations between SSRT and go-RT, such as repeatedly demonstrated for stop-signal probability that affects go-RT but not SSRT (Kenemans, 2015; Lansbergen et al., 2007). A relevant study in this respect was reported by Chang et al. (2017). Using an estimate of subjective stop-signal probability, which was significantly associated with subsequent stop success, these authors reported a significant association between this subjective probability and theta power preceding the go stimuli (Chang et al., 2017). In contrast, pre-go theta was not associated with go-RT. However, a more direct test of this dissociation between stopping and slowing would involve examining theta power as a function of subsequent go-RT, which was a further aim of the current study.

In the present study, we investigated EEG FM theta power in two separate experiments utilizing stop-signal paradigms in healthy individuals. Both experiments are based on the same stop-signal paradigm. This paradigm consists of regular go trials, in which participants are instructed to respond by pressing a button, and infrequent stop trials, in which participants are required to withhold their response. By comparing frontal theta power preceding failed versus successful stop trials, we aim to test whether frontal theta is predictive of inhibition success. We hypothesized that proactive cognitive control manifests in frontal theta power preceding a countermanding go-stop event. Thus, we expect higher frontal theta preceding successful vs failed stop events. Additionally, if frontal theta power is specifically indicative of increased proactive inhibitory control, but not proactive slowing, we expect overall theta power preceding go-stop events to be associated with shorter average SSRT values but not with longer go-RTs. More specifically, higher theta power preceding go trials with long compared with short RTs would also translate into an association between higher theta power and successful stopping, because a longer go-RT promotes stopping success.

Lastly, we compare conditions of auditory versus visual stop signals. Our expectation is that theta power related to proactive control is a higher-order, supra-modal mechanism; therefore, we expected no difference in it between sensory modalities (Kenemans et al., 2023).

## 2 Method

The first experiment concerned an additional analysis of already published data (Kenemans et al., 2023), as a first exploration of the prospective association between FM theta and stopping success. To this aim, we utilized the data from a stop-signal task with an auditory stop-signal only.

The second experiment was acquired specifically to expand upon the frontal theta comparison of the first experiment, by including a larger sample and a version of the stop-signal task that includes both auditory and visual stop-signal varieties. Both experiments were based on the same version of the stop task; minor differences in procedures between experiments will be indicated where relevant.

### 2.1 Participants

For experiment 1, EEG and stop-signal task data were taken from an existing sample previously described in Kenemans et al. (2023). This sample included 28 participants (mean age = 22.9 years, SD = 3.3; n = 19 females). For rationale for this sample size, see Kenemans et al. (2023). The effect size for this sample was entered in G*power version 3.1.9.7 to estimate the sample size in experiment 2, giving a lower bound of 35 participants.

For experiment 2, EEG and stop-signal task data were acquired for 52 healthy volunteer participants (mean age = 24.2 years, SD = 4.5; n = 31 females). Since in this study additional statistical tests were planned, especially with respect to across-participant correlations, the sample size was increased with an arbitrary 50% relative to the estimated lower bound calculated above.

All participants signed an informed consent form and reported normal hearing and normal or corrected‐to‐normal vision. Participants were paid 20 Euros for their participation. The study was approved by the local ethics committee of the faculty of Social and Behavioral Sciences of Utrecht University.

### 2.2 Procedure

The procedure was identical for both experiments. Participants read and signed the informed consent and provided demographical information before the EEG cap and EEG compatible in-ear headphones (13 or 10 mm based on the participant’s ear size, Etymotic Research, Inc.) were mounted. Participants were seated in a dimly lit room, with their head on a chin-rest 60 cm centered in front of the computer screen. After receiving the instructions, participants’ performed the stop-signal task (see details below) for approximately 35 minutes (plus a 5-minute break halfway) during which EEG was recorded.

### 2.3 Stop-signal task

The stop-signal task (SST) consisted of a two‐choice response task (Kenemans et al., 2023). Participants had to discriminate between two go‐stimuli: the letters “X” and “O” (visual angle for both letters, 1.6° x 1.6°), by pressing the left or the right button with the left or the right index finger, respectively. The mapping of “X” and “O” to either left or right was counterbalanced over blocks and conditions.

A single total trial duration was always preset at 1,500 ms, rounded up to the start of the next cycle in the screen-refresh rate (60 Hz). A fixation cross remained on screen for the entire duration of the trial, as well as during the Inter-Trial-Interval (ITI). The go stimulus was then presented first, for 150 ms, centrally 0.5° above the fixation cross. On 25% of the trials, a stop signal was presented after the go-signal, following a variable Stimulus Onset Asynchrony (SOA, see below for detailed description). For each subject, two varieties of the task were presented using either an auditory or visual version of the stop signal. In the visual condition, the stop signal consisted of a screen with a red background, presented for 150 ms. In the auditory condition, the stop signal consisted of a 1,000 Hz tone (72 dB), presented for 150 ms in both ears via in-ear headphones. After the go- and potential stop signals were presented, the ITI was calculated to fill the remainder of the trial to make up the total duration of 1,500 ms. Figure 1 presents an overview of the trial structure.

**Fig. 1.**
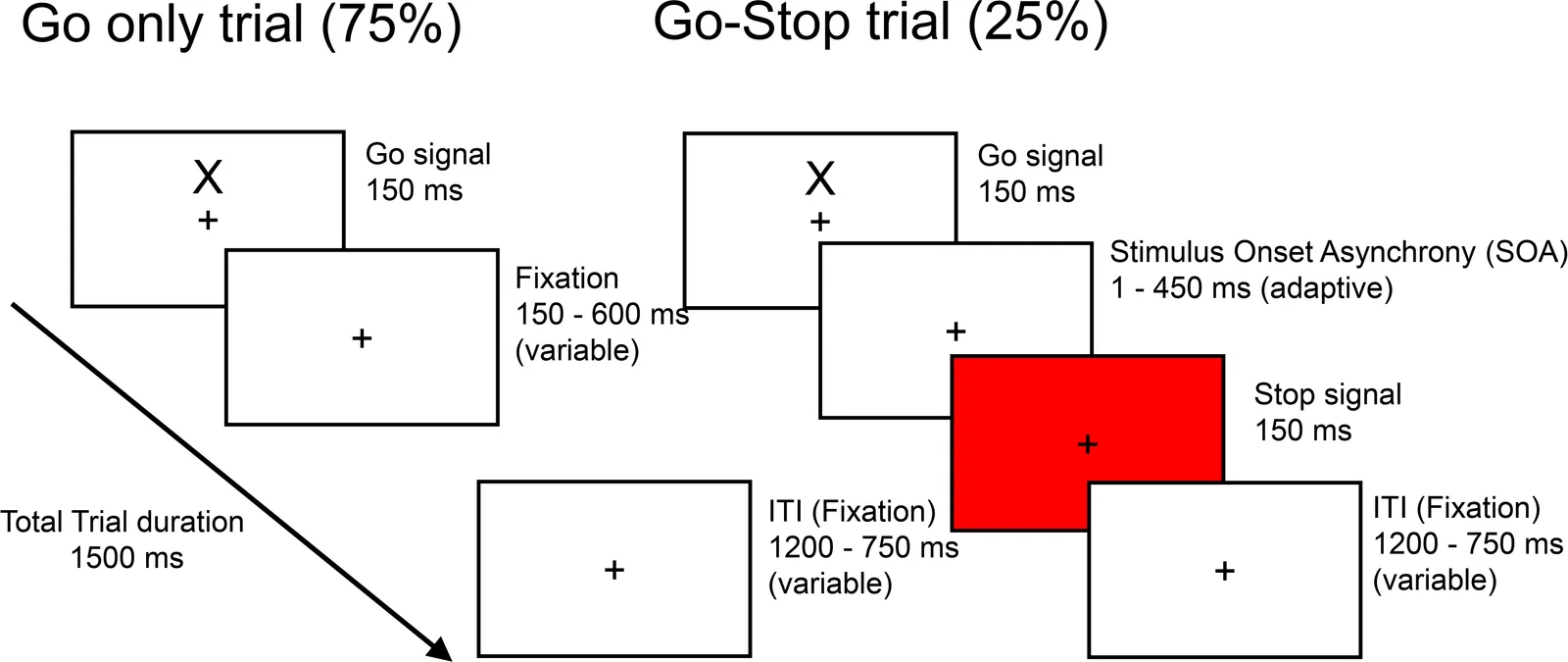
An example of a go (left) and a stop trial with a visual stop signal (right).

Experiment 1 (auditory stop signal) consisted of a total of nine blocks, this included three practice blocks and six experimental blocks, divided over two task conditions representing the two possible response mappings. In one condition, an “X” instructed a left hand button press, an “O” instructed a right press, with the mapping reversed in the other condition. This mapping was counterbalanced over participants. The first practice block consisted of 24 go trials, and was also used to establish a baseline reaction time on go-trials (go-RT). For each task condition, a second practice block was included consisting of 128 trials, this included 32 stop trials. Across the 6 experimental blocks, a total of 6 × 128 = 768 experimental trials were included per subject, including 192 stop trials.

Experiment 2 (auditory and visual stop signals) consisted of a total of 13 blocks, this included 5 practice blocks and 8 experimental blocks, divided over 4 task conditions (2 stop-signal modalities x 2 response mappings). The first practice block consisted of 24 go trials, and was also used to establish a baseline reaction time on go-trials (go-RT).

Next, the three blocks per condition were presented (one further practice block and two experimental blocks). All participants were presented with both visual and auditory stop conditions. The different conditions were blocked and counterbalanced, so that the “X” and “O” were mapped to left/right hand, respectively, in a counterbalanced way. Each block consisted of 128 trials. The first block of each condition was a practice block, used to estimate the average SOA. Practice blocks were not included in the eventual analyses, so only the 8 experimental blocks were analyzed, for a total of 8 × 128 = 1,024 trials per subject; this included 25%, or 256, stop trials. Halfway through the experiment, participants were offered a short 5-minute break.

As mentioned above, for both experiments, the SOA was adjusted during the experimental blocks to adapt to the individual participants’ performance. If participants managed fewer than 13 successful stops, they were requested to slow down; if they managed more than 19 successful inhibitions, they were requested to speed up. The SOA between the onset of the go and the onset of the stop stimulus was set at 250 ms at the start of the experiment, and was adjusted using a tracking algorithm during each practice block, by increasing the SOA with 50 ms after each successful stop, and shortened with 50 ms after each failed stop trial, leading to an adapted difficulty for every subject. The resulting final value of each practice block (with a minimum possible SOA of 250 ms) was set to be the average SOA for the subsequent experimental block. After each experimental block, the average SOA was further updated by subtracting the observed successful stop rate from the expected 50%, and adjusting the new SOA by 2x the difference in percentage converted to milliseconds (de Jong et al., 1990), with a minimum value of 250 ms and a maximum of 600 ms. Within each block, the SOA was subsequently jittered between + and -99 ms around the average over trials (uniform distribution of jitter values). This method of SOA adjustment should lead to an average successful stop rate of 50% for all participants for the experimental blocks (Verbruggen et al., 2019). If, despite these adaptive mechanisms, participants still scored outside of the 30–80% successful stop rate for any given block, subsequent calculations of the SSRT can be considered unreliable, and these blocks were excluded from further analyses. Note that the original motivation for this jitter approach was to be able to use the Adjar-level-2 procedure to separate go and stop Event-Related Potentials (ERPs). However, this is not relevant in the current study where we considered only pre-go EEG power.

### 2.4 Performance analysis

For both experiments, stop-signal response times were analyzed following the procedure outlined by Verbruggen et al. (2019). First, per block, the proportion of successful stops was calculated. Subsequently, all go-RTs and omissions (for which RT was set at a fixed value of 1,500 ms) were rank ordered from shortest to longest. Then the inverse stop rate (=percentage failed stops) was used to identify the corresponding sorted-RT-distribution percentile (e.g., with an inverse stop rate of 45% and 120 sorted go-RTs, the (0.45 × 120)th go-RT was taken). The average SOA for that block was subtracted from the selected RT to estimate SSRT for that block. Subsequently, SSRTs were averaged across blocks. Mean RTs per condition and per participant were estimated by averaging valid go responses only (so excluding omissions, choice errors, and RTs shorter than 200 ms or longer than 1,500 ms).

### 2.5 EEG data acquisition

EEG signals were recorded with the Active‐Two system (Biosemi, Amsterdam, The Netherlands) with 64 Ag–AgCl electrodes. Recording electrodes were placed according to the 10/10 system. Four flat-type electro-oculogram (EOG) electrodes were placed: above and below the left eye and at the outer canthi of both eyes. Offline reference electrodes were placed at the left/right mastoid bones. EEG signals were online referenced to the Common Mode Sense/Driven Right Leg electrode, sampled at 2,048 Hz and online low pass filtered at DC to 400 Hz.

### 2.6 EEG power analyses

Raw EEG signals were analyzed offline using Brain Vision Analyzer (BVA) 2.0 (Brain Products GmbH). EEG data were resampled to 64 Hz (after adequate low-pass filtering), re-referenced to the mastoid reference electrodes, and high-pass filtered (0.5 Hz cutoff). Data were divided into 1-second segments (950 ms before to 50 after a go stimulus that was followed by a stop signal). Automatic ocular correction was performed using the EOG channels (Gratton et al., 1983). After ocular correction, automatic artifact rejection was used (maximum allowed absolute difference between two values: 100 μV, lowest allowed activity within a 100 ms interval: 0.5 μV). Trials containing a stop signal were split for successful and for failed stops.

To validly compare theta power preceding both failed and successful stop trials, an equal number of failed and successful stop trials has to be included in the analyses. Out of the condition with the highest number of trials, the redundant number of trials was randomly selected and removed. Across all participants, this led to the inclusion of an equal number of 96 trials on average (SD = 14, range = 57–121), of both failed- and successful stop events.

Subsequently, spectral periodograms were estimated (per trial per condition per participant), using a fast Fourier transformation (FFT; Hanning window: 10%) and averaged across trials per condition to obtain power spectra, from which theta power values were extracted (Schutte et al., 2023; average power across 3–8 Hz).

### 2.7 Analysis of theta power as a function of stop success

To compare the differences in theta power preceding failed vs successful stop trials for experiment 1, a paired-samples t-test was employed. For experiment 2, to compare both the auditory and visual conditions of the SST, a two-factor within-subject ANOVA was performed, including stop success (failed/successful) and modality condition (visual/auditory) as within-subject factors.

To analyze the relationship between FM theta values in the different conditions and stop-signal task performance, a multiple linear regression model was performed consisting of theta power (averaged across failed and successful events) as the dependent variable and mean SSRT and mean go-RT as numerical predictor variables. For experiment 2, an additional within-subject factor was added for modality (Auditory/Visual).

### 2.8 FM-theta specificity

A further analysis concerned the oscillatory specificity of the FM theta effect. To this end, we analyzed stop success for frequencies ranging from 1 to 15 Hz, with 1 Hz resolution. In this way, the extent to which these effects were specific for the 3–8 Hz band was explored.

### 2.9 Stopping success and SOA

To zoom in on FM theta differences between failed and successful stops, we reasoned that FM theta may be especially associated with stop success when stopping is difficult (as with long SOAs) compared with when it is easy (as with short SOAs) but still fails. Hence, as an additional exploratory analysis, stop events were separated based on the length of their SOA, grouping all the trials with below-median SOAs (short) and above-median SOAs (long). We then compared successful stop trials with a long SOA (difficult but successful) with failed stop trials with a short SOA (easy but failed), using a paired-samples t-test. We expected that if FM theta is reflective of proactive inhibition, the mean difference in theta power between failed and successful trials should be amplified in this secondary analysis.

### 2.10 Time–Frequency analysis (TFA) for stop-success-related EEG

We next addressed how FM theta power evolves over time (preceding go-stop trials). First, for go-stop trials, a wavelet time–frequency analysis (TFA) was performed, using BVA’s wavelet function. For an initial time–frequency plot, values were extracted for the frequency range of 1 to 30 Hz, using a repetition of 3 cycles (Morlet parameter), a temporal resolution of 173.6 ms, and uniform scaled normalization. The resulting values were used to construct a participant-averaged time–frequency plot (TFP) for the FCz electrode.

To focus on theta-power, single values with a central frequency of 5.5 Hz and half-Gauss borders at 3.7 and 7.3 Hz were extracted as a function of time (from -850 to -50 ms in equal steps; the first and last 100 ms of the -950 to 50 ms window were excluded to avoid uncertainties associated with tapering). This was done separately for successful versus failed stops, and separately for auditory versus visual stop-signal modality. These values were analyzed in a Time × Stop success × Modality design to capture the more precise time course of theta power as associated with successful stopping. Note that the analyzed power values are absolute, not baseline corrected.

Based on visual inspection of the TFP, the same analysis was performed for FCz slow-alpha power, between -450 and -50 ms, where slow alpha is defined as the slowest alpha waves between 8 and 10 Hz. To extract these slow-alpha values, a central frequency of 8.3 Hz and Gauss borders of 5.5 and 11 Hz were used.

### 2.11 Comparison between stop-success-based and go-RT-based trial selection

Subsequently, TFA was applied with the same settings to the 1-second EEG segments preceding slow versus fast go trials (separated based on median split). Single theta power values were extracted for the time period as identified in the stop-related time course analysis (this turned out to be -850 to -550 ms, where theta values were at maximum). Likewise, single slow-alpha power values were extracted for the same time and frequency ranges as in the stop-related analysis.

Finally, for both theta and alpha, a combined design was constructed to comprehensively test for any difference between stop-related and go-related effects. Specifically, this design included the factors Trial-Selection (stop versus go), trial Outcome (success/ slow versus failed/ fast), and Modality (auditory versus visual). As a comprehensive evaluation of the Trial-Selection effect should also take into account topographical specificity, contrasts to model this were additionally included in the design. Specifically, to model topographical laterality and anteriority, theta and alpha values were extracted from 28 sensors (7 laterality levels, F5, F3, F1, Fz, F2, F4, F6; repeated with the same row for F, FC, C, and CP electrodes for 4 anteriority levels). The difference score values in power from the three first within-subject factors were convolved with a polynomial-term matrix representing 0-order linear, quadratic, and cubic trends across both laterality and anterior–posterior sites. To control for type-I error in evaluating topographical effects, the critical p-value was set at 0.003 (=0.05/16), as there were 4 × 4 polynomial contrasts for the combined anteriority and laterality factors.

## 3 Results

### 3.1 Performance

Performance data on the SST for both experiments are depicted in Table 1. In experiment 2, a significant effect of stop-signal variety on SSRT is observed (t(47) = -3.579, p < 0.001). As discussed in the Introduction, there is no straightforward relationship between SSRT and mean go-RT, with numerous examples of single dissociations. Consistently, in the present study, we also found that SSRTs were longer for visual than for auditory stop signals, whereas stop-signal modality had no effect on go-RT (see Table 1); this is a direct replication of the finding reported in Kenemans et al. (2023).

**Table 1.** Behavioral metrics for successful trials for all experiments, reported by auditory and visual modality where relevant.

	Experiment 1	Experiment 2	
	Visual	Auditory	Visual
SSRT (ms)	213	173*	194*
Go-RT (ms)	665	624	647
RT var (ms)	113	140	144
SOA (ms)	450	441	425
Stop rate (%)	49	50	56

* indicates a significant effect (p < 0.05).

SSRT = stop-signal reaction times; Go-RT = mean reaction time on go trials; RT var = mean standard deviation of go-RT; SOA = stimulus onset asynchrony between go and stop signals; Stop rate = mean % of successful stops.

Given that this manuscript is about the proactive theta power before a trial onset, an additional set of metrics is depicted in Table 2, indicating the nature of the previous trial for both successful and failed stop events for both experiments. Paired-samples t-tests indicate that previous trial go-RT was significantly longer for successful than for failed stop trials (t(28) = 8.1, p < 0.001; t(47) = 10.03, p < 0.001 for experiments 1 and 2, respectively), but no other significant differences were observed. SOAs for the current trials were also compared between failed and successful stop trials, and were also not significantly different.

**Table 2.** Depicted are the previous trials identity metrics, indicating what happened on the trial before a successful or failed stop for each experiment, split for successful and failed stop trials.

	Experiment 1				Experiment 2			
	Before failed stop		Before successful stop		Before failed stop		Before successful stop	
Previous trial identity	Mean	SD	Mean	SD	Mean	SD	Mean	SD
SOA (ms)	436	96	466	70	424	129	451	104
Succ inhibitions (%)	14	3	10	2	14	4	13	3
Failed inhibitions (%)	16	4	11	3	14	4	10	2
Go trials (%)	70	3	79	3	72	3	77	4
Go-RT (ms)	658*	151	596*	133	687*	120	613*	106

* indicates significant differences between trials preceding successful versus failed stops.

SOA = stimulus onset asynchrony between go and stop signals; % succ/failed inhibitions = mean % of successful and failed stop trials before the current stop trial; % go trials = % of go trials before current stop trial; Go-RT = mean RT on go trials before each type of stop trial.

### 3.2 Analysis of theta power

For experiment 1, theta power was maximal at the FCz electrode in all conditions. In contrast, in experiment 2, the maximal theta power was observed at Fz, in both visual and auditory conditions. Therefore, for both experiments, we quantified FM theta power as the average spectral power across Fz and FCz; this also partially reflects the relevant literature that reveals a mixture of FCz and clusters of frontal electrodes (e.g., FCz: Chang et al., 2017; Mazaheri et al., 2014; Messel et al., 2021; Fz: Adelhöfer et al., 2021; Mazaheri et al., 2010; clusters: Cooper et al., 2017). Mean values for FM theta power for failed and successful stop trials in auditory and visual conditions of the stop-signal task are depicted in Table 3. Group averages of theta power scalp distribution are depicted in Figure 2.

**Fig. 2.**
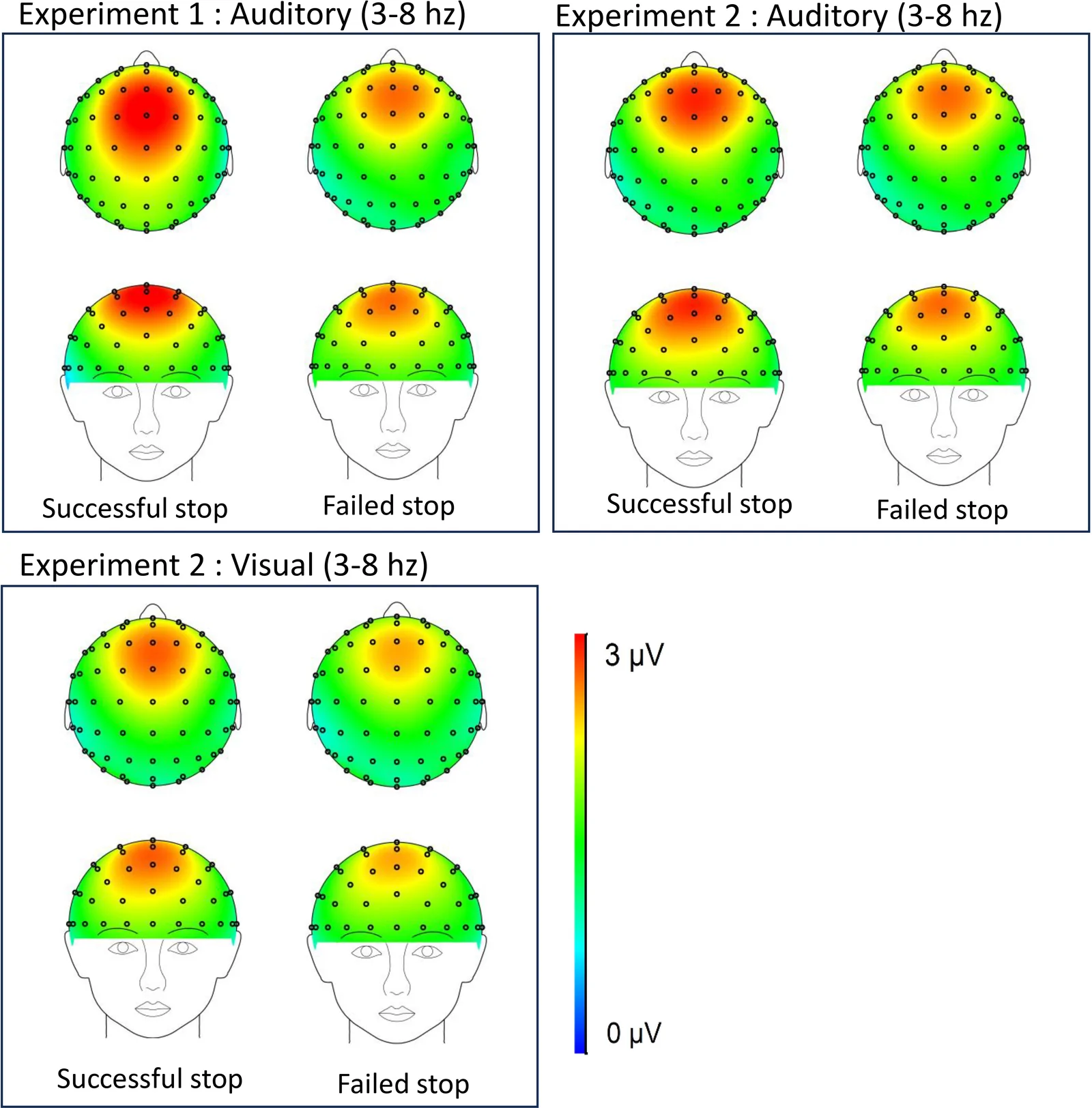
Topography of average theta power preceding successful and failed stop trials for experiments 1 and 2. For experiment 2, auditory and visual conditions are shown separately.

**Table 3.** Mean FM theta power values (Fz and FCz averaged) preceding both the failed and successful stop trials for both experiments, reported by auditory and visual modality where relevant.

	Experiment 1		Experiment 2			
	Visual		Auditory		Visual	
Theta power (µ^2^)	Mean	SD	Mean	SD	Mean	SD
Failed stops	3.89	2.2	2.6	1.5	2.5	1.1
Successful stops	4.39	2.7	2.8	1.8	2.7	1.4

For experiment 1, the paired-samples t-test comparing FM theta power between failed and successful stop trials indicates significantly lower theta power preceding failed trials than preceding successful trials, see Table 4.

**Table 4.** Model summary of the within-subject analysis comparing average FM theta power preceding both failed and successful stops for both experiments.

	Experiment 1			Experiment 2		
Theta power (µ^2^)	t(1,27)	d	p	F(1,47)	d	p
Failed/successful stops	2.666	0.49	0.013*	11.141	1.47	0.002**
Auditory/visual				3.041	0.19	0.08
Failed/successful* Auditory/visual				0.01	0.03	0.93

* indicates a significant effect (*p < 0.05; **p < 0.01).

In experiment 2, auditory and visual conditions are added as an additional factor.

In experiment 2, the results from the two-way within-subject ANOVA (Stop success x Modality) revealed a main effect of stopping performance (see Table 4; Fig. 3). FM theta power preceding failed trials was lower than preceding successful trials for the auditory stop condition as well as for the visual stop condition. There was no significant difference in theta power between auditory and visual stop-task conditions, nor was there a significant interaction between stop-task condition (auditory/visual) and stopping performance.

**Fig. 3.**
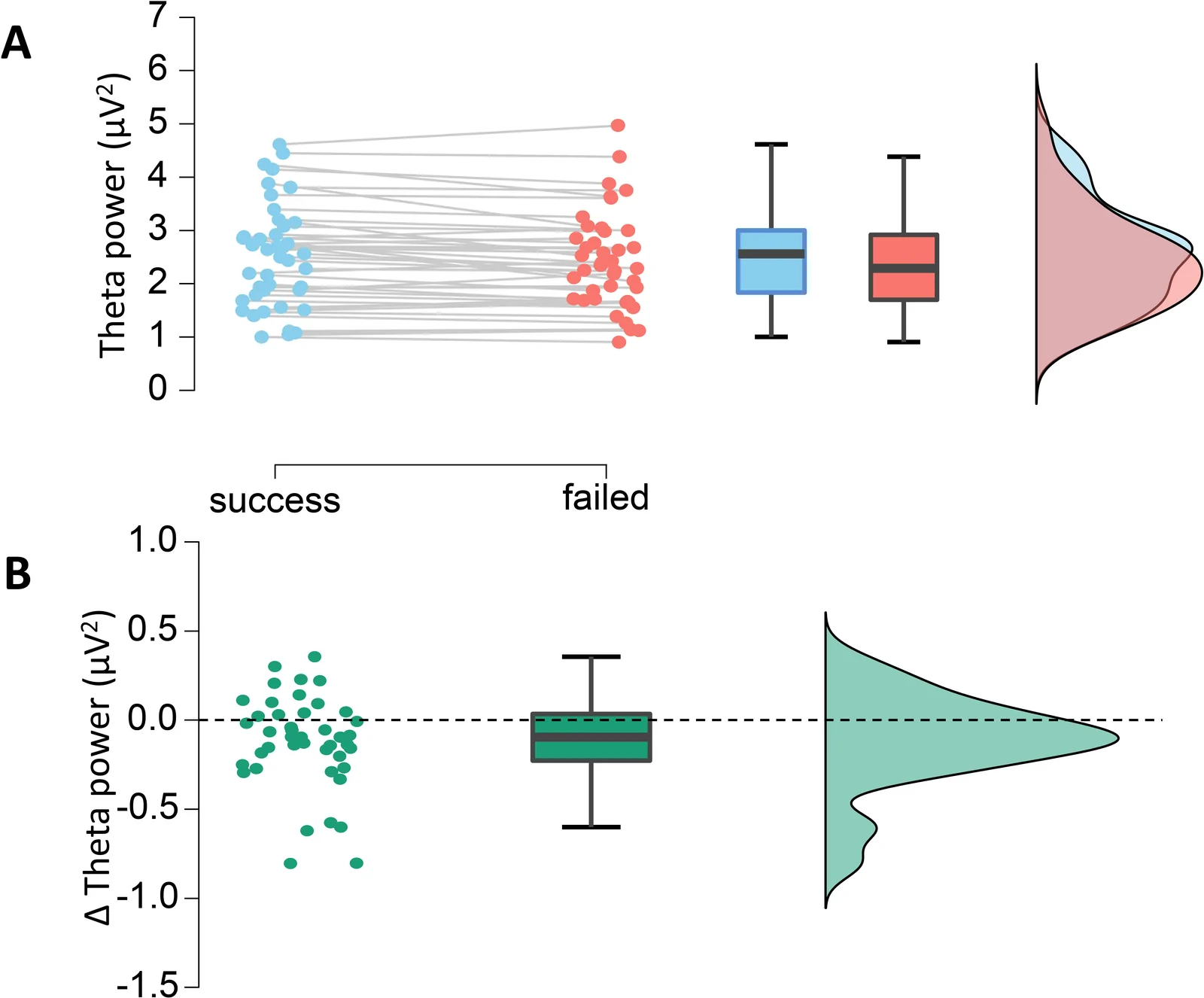
FM theta comparison for experiment 2. (A) Mean frontal theta preceding failed and successful stop trials. Points represent theta power preceding failed/successful stop trials for individual participants, bar graphs and density plots show the average over all participants. (B) The difference score in mean frontal theta power preceding failed versus successful stop trials per subject (points), as well as averaged (bar graph and density plot).

For a first perspective on how specific the relationship between SSRT and stop success is, the across-participant associations between SSRT and go-RT on the one hand, and FM theta on the other, were evaluated in a multiple regression model with SSRT and go-RT as predictors for FM theta power. Both associations were significant only for experiment 2. For SSRT, the partial (RT-controlled) correlation was -0.28 (p < 0.001); for go-RT it was 0.17 (p < 0.05). Both the negative correlation with SSRT and the positive correlation with go-RT are consistent with an association between theta and both proactive inhibition and proactive slowing, respectively.

### 3.3 FM theta specificity

To shed light on the specificity of the stop-success effect for theta, all frequencies ranging from 1 to 15 Hz (step size 1 Hz) were tested for a stop-success effect averaged across the whole time segment preceding stop events (see Fig. 4) at Fz and FCz averaged.

**Fig. 4.**
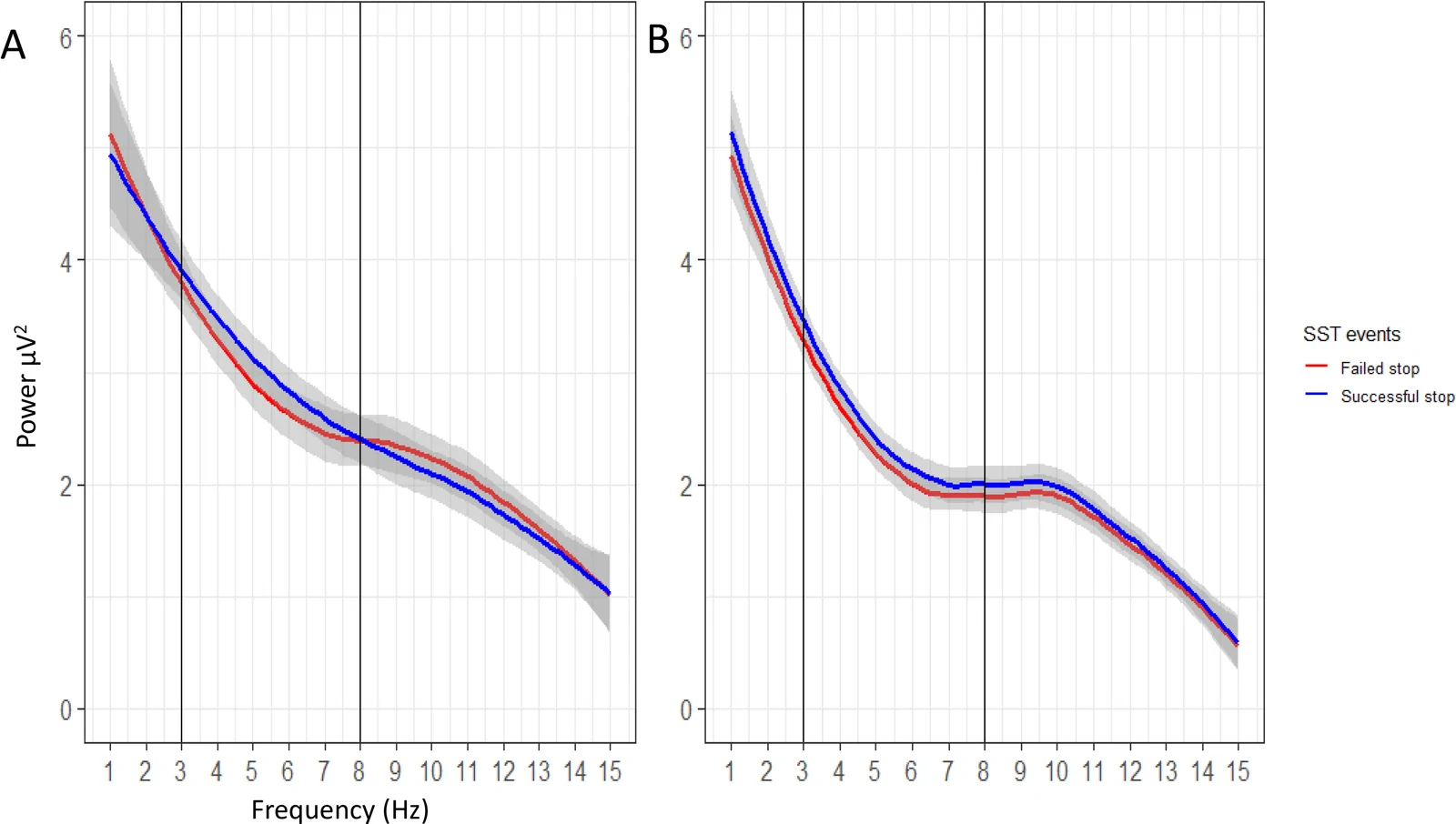
Absolute power spectra over frequencies from 1 to 15 Hz averaged over the 1,000 ms preceding both failed (red) and successful (blue) stop events in experiment 1 (A) and experiment 2 (B), averaged across Fz and FCz.

We found significantly larger power (success versus failed) at 3, 6, and 9 Hz at Fz and FCz averaged in experiment 1 (p < 0.05). This indicates that, in this sample, the stop-success effect was specific for theta. In experiment 2, we found significantly larger power (success versus failed) at 2, 3, 5, 7, 9, 10, and 13 Hz. To project this on the classical frequency bands, the difference was tested for the average power between 1 and 3 (delta), 4 and 8 (theta), and 9 and 13 Hz (alpha). Larger theta power for success than for failed was found for theta and for alpha (p < 0.05), not for delta. This indicates that the stop-success effect was only partly specific for theta.

### 3.4 Stopping success and SOA

In experiment 1, SOA split analysis for stop trials indicated that FM Theta power (Fz + FCz averaged) preceding failed trials with a short SOA was lower than preceding successful trials with a long SOA (M_short_ = 3.74 ± 2.1; M_long_ = 4.67 ± 3.6; t(28) = 2.9; p < 0.05, d = 0.35; see Fig. 5). In contrast, the difference between failed trials with a long SOA and successful trials with a short SOA was not significant (t(28) = 0.64; p = 0.756).

**Fig. 5.**
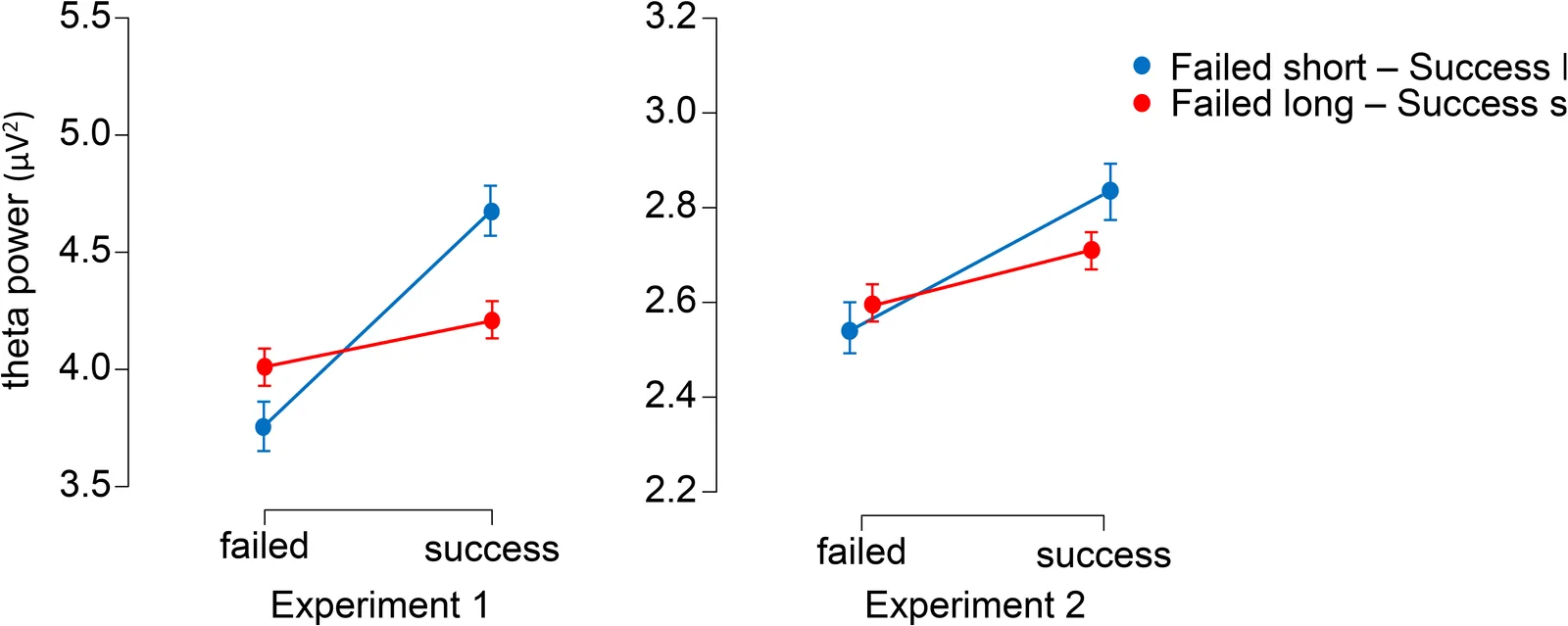
Mean FM theta values for the comparison between failed stop trials with a short SOA (difficult trials) and successful stop trials with a long SOA (easy trials) and vice versa, for experiments 1 and 2.

In experiment 2, the same results were obtained, with FM theta power preceding failed trials with a short SOA was lower than preceding successful trials with a long SOA (M_short_ = 2.54 ± 1.3; M_long_ = 2.84 ± 1.7; t(47) = 2.9; p < 0.05, d = 0.18). These findings are in line with the expectation that the mean theta difference between failed stop trials with a short SOA and successful stop trials with a long SOA would be greater than the same differences without separating short versus long SOAs (compare with Table 3).

### 3.5 Time–frequency analysis (TFA) for stop-success-related EEG

Figure 6 shows time–frequency plots for the successful/failed stop contrast. Figure 7 shows the extracted single FM theta values as a function of Time, Theta power as well as the Stop-success effect peaking between 850 and 550 ms before go onset, irrespective of Modality. The Time x Stop success x Modality ANOVA revealed main effects of Time (F(7,41) = 4.3, p < 0.005) and Stop success (F(1,47) = 12.7, p < 0.001), but no main effect of Modality or any significant interactions. Additionally, the 8 time segments were separately tested for Stop-success effects, which were significant at p < 0.007 for -850 to -550 ms, with all p-values exceeding 0.05 for later windows.

**Fig. 6.**
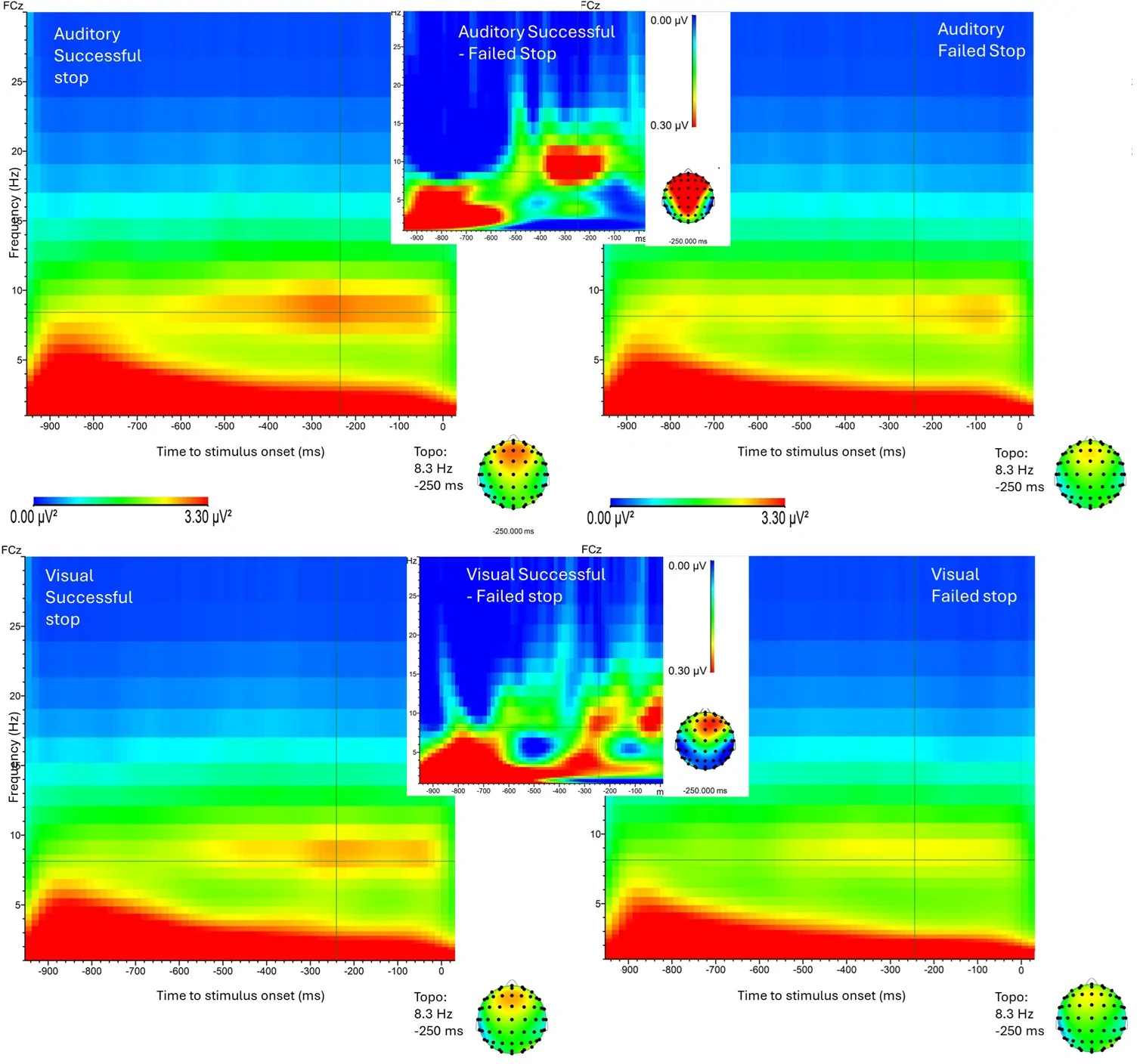
Time–frequency plots for absolute oscillatory power across the 1,000 ms preceding successful versus failed stop trials, at the FCz electrode. Both plots based on experiment 2 only. Plots shown separately for auditory and visual modalities. Red values indicate higher power, blue values indicate lower power. Time–frequency plots for successful trials are shown left, failed trials are shown right. The top-middle inlays show the difference maps between successful and failed trials, with separate power scale and topography map included.

**Fig. 7.**
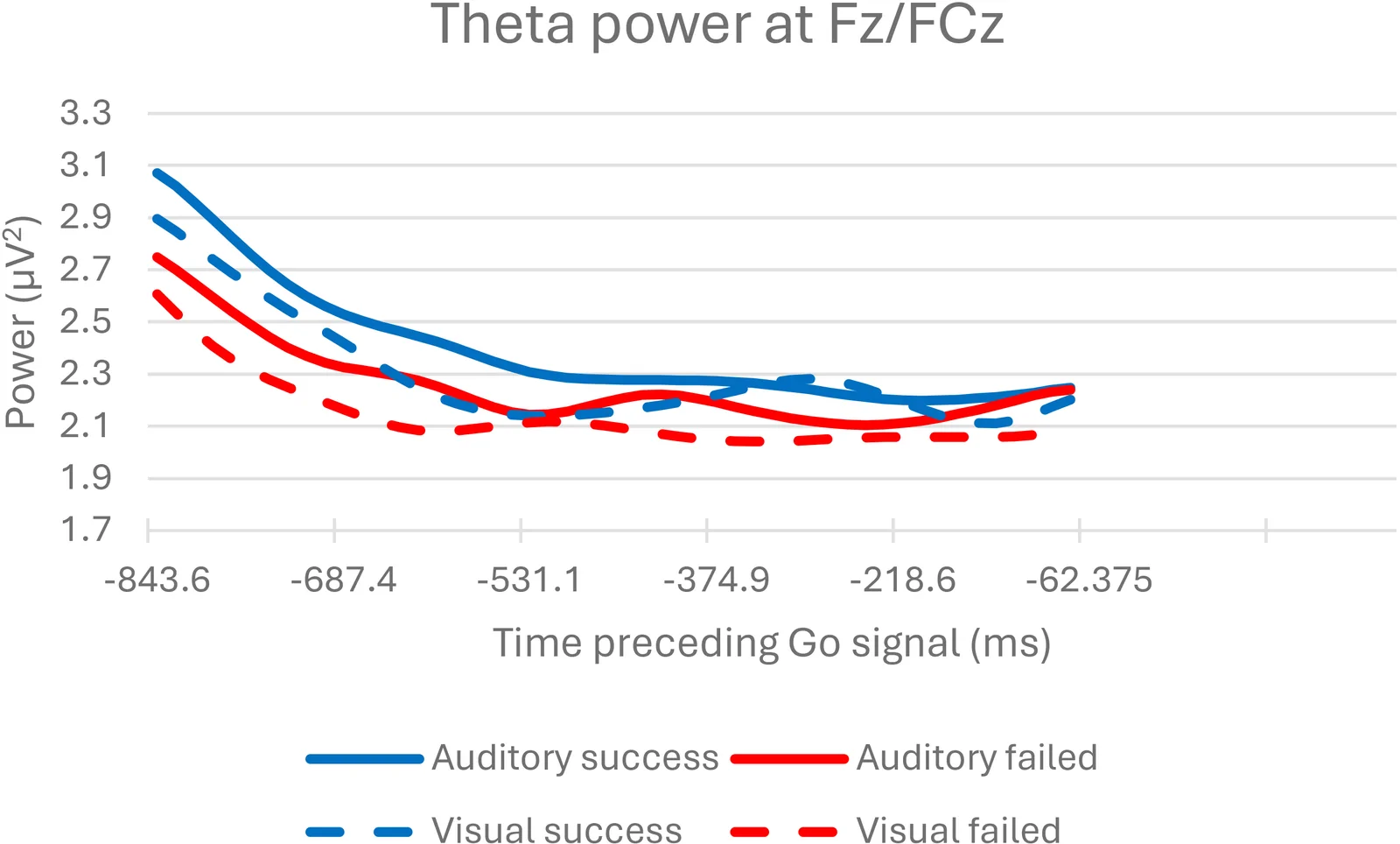
Mean theta power over time (averaged over Fz and FCz locations) before Go-signal onset, as a function of stop-signal modality (Auditory/Visual), and stopping success (Success/Failed).

Figure 6 also suggests a pattern for alpha power featuring a topographical distribution similar to that of theta (see topographic inset), as well as an association with time in the second half of the pre-go epoch, and also a difference between stop-success conditions. Hence, an additional analysis was added here to further examine the nature of alpha power before the stop trials. We refer to this as “slow-alpha” power because the maximum power seems to center around 8 Hz (see Fig. 6). Slow-alpha power (Fz/ FCz) with a central frequency of 8.3 Hz and Gauss borders of 5.5 and 11 Hz was stronger for the second half than for the first half of the epoch (F(1,47) = 8.7, p < 0.01, and stronger for subsequent successful than for failed stops (F(1,47) = 19.4, p < 0.001). Conspicuously, the stop-success effect was also more pronounced for auditory than for visual stop signals (F(1,47) = 12.4, p < 0.001).

### 3.6 Comparison between stop-success-based and go-RT-based trial selection

As to theta power preceding slow versus fast go-RTs, Figure 8 visualizes the FFT results for this comparison. Figure 9 shows the corresponding TFPs. The comprehensive analysis for theta power (-850 to -550 ms) revealed a significant main effect of Outcome at F(1,47) = 17.7, p < 0.001), reflecting stronger theta power before both successful stops and slow go trials. This was not qualified significantly by effects of Trial Selection, Modality or their interaction (all p > 0.1). Across modalities then, there was no difference between the association of theta with stop success, and that of go-RT, suggesting the former association can be statistically explained by the latter.

**Fig. 8.**
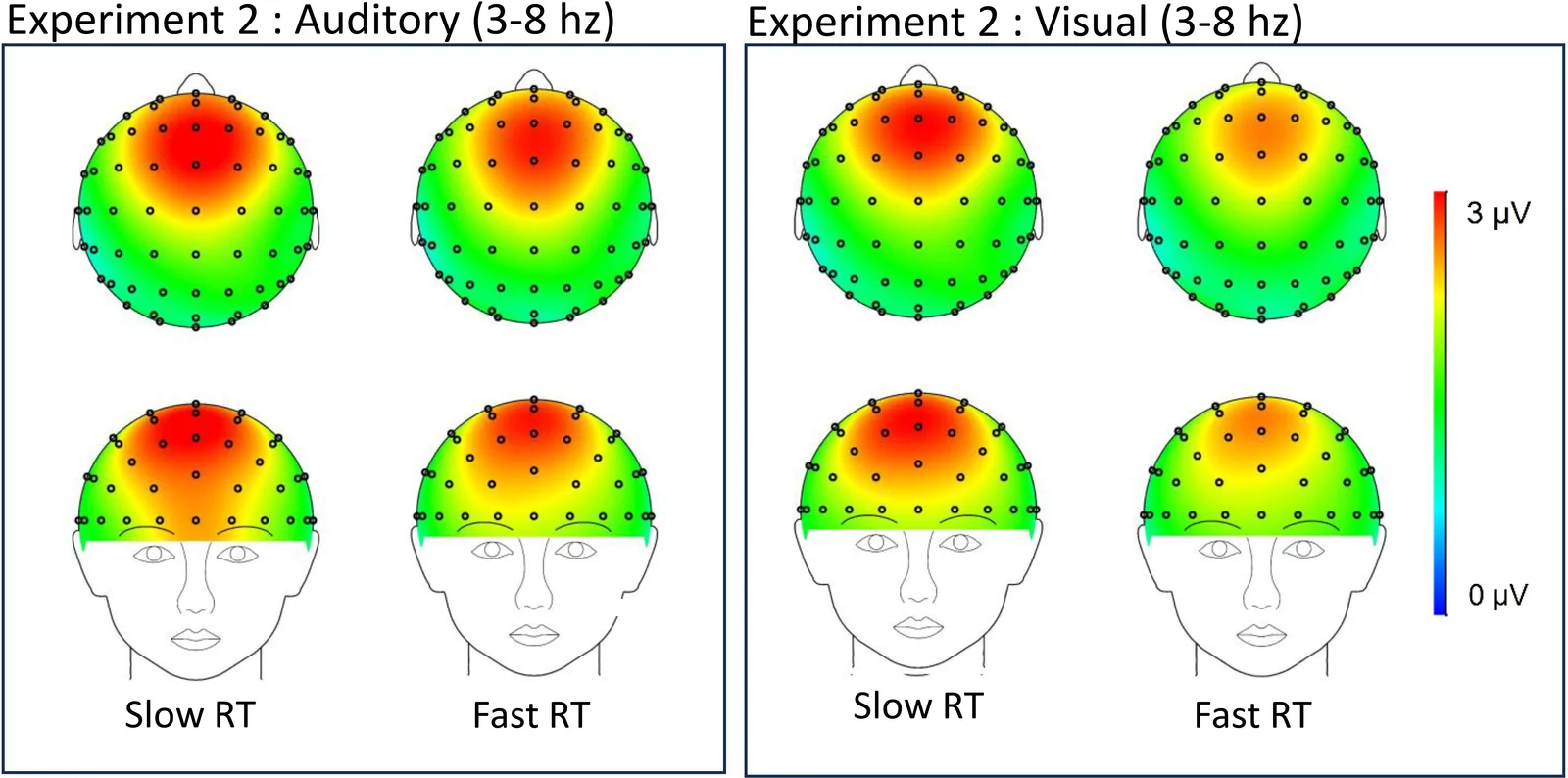
Topography of average theta power preceding Slow and Fast go trials for experiment 2, auditory and visual conditions are shown separately.

**Fig. 9.**
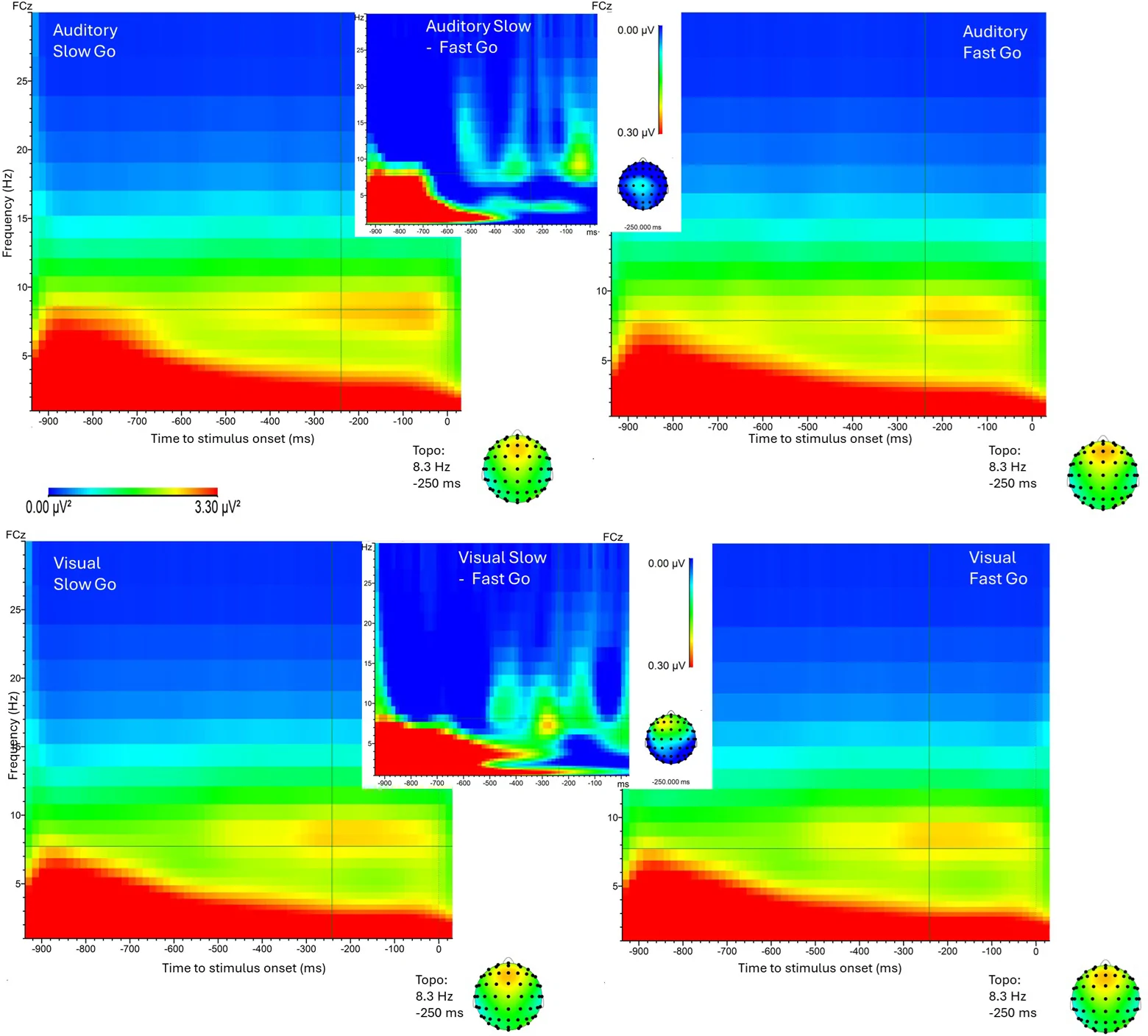
Time–Frequency plots for absolute oscillatory power across the 1,000 ms preceding slow versus fast go trials. Plots based on experiment 2 only. Comparisons shown separately for auditory and visual modalities. Red values indicate higher power, blue values indicate lower power. Time–frequency plots for slow go-trials are shown left, fast go-trials are shown right. The top-middle inlays show the difference maps between slow and fast trials, with separate power scale and topography map included.

As to topography, at p < 0.003 there was only an interaction between the effects of Outcome, Laterality (quadratic), and Anteriority (quadratic; F(1,47) = 10.2). As shown in Figure 10, this confirms the clear FM distribution of the association between theta and outcome (quadratic-laterality effect); it also confirms (quadratic-anteriority effect) that this FM signature was most pronounced at the FC line, followed by the F and the C line.

**Fig. 10.**
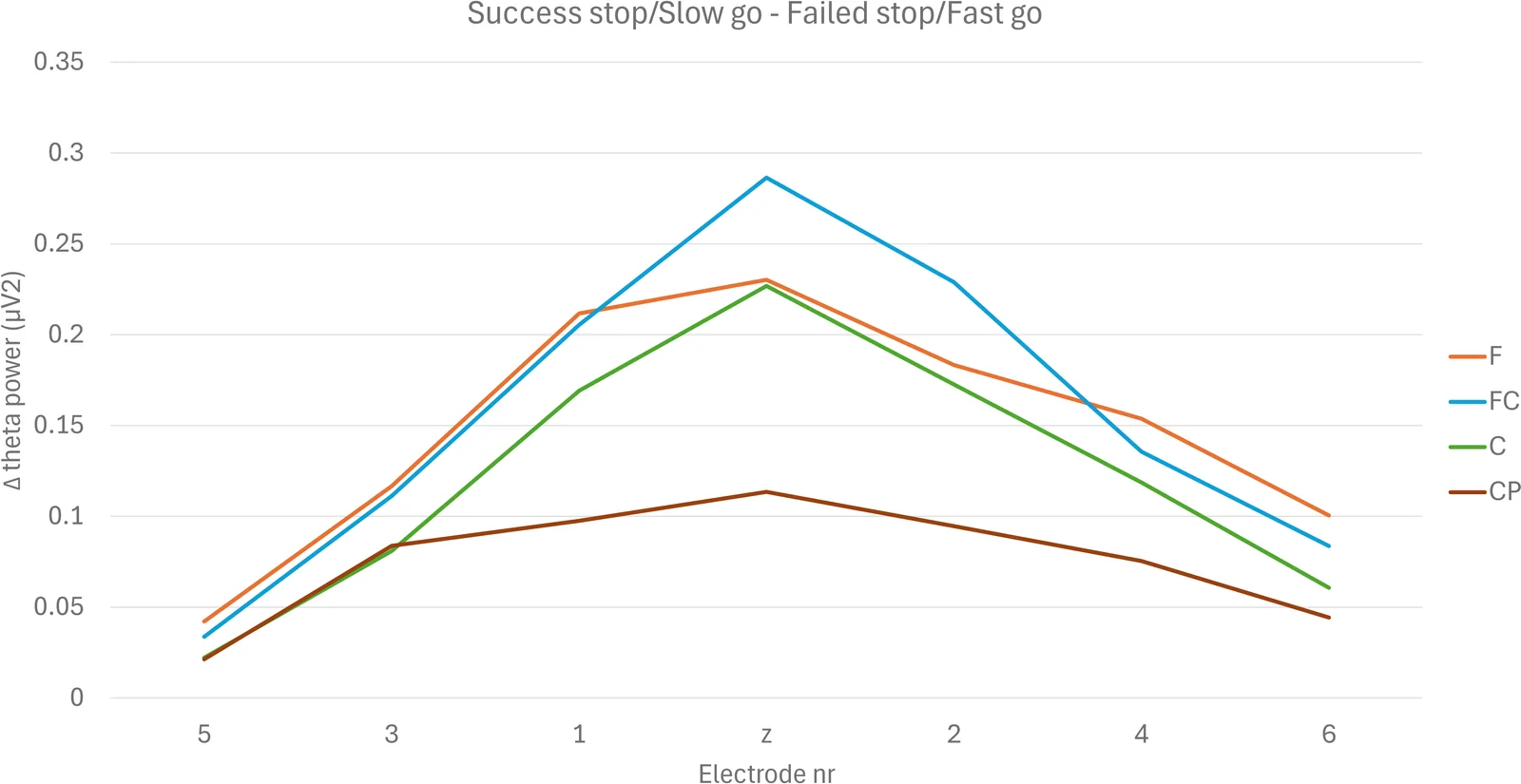
Theta power difference between successful stop and failed stop events or slow go and fast go events, averaged over both contrasts. x axis represents electrode locations X5, X3, X1, Xz, X2, X4, X6; where X = F, FC, C, CP.

For slow-alpha power, a significant effect of Outcome (F(1,47) = 7.6, p < 0.01) was qualified by an interaction between Outcome and the Trial-Selection factor (F(1,47) = 7.6 (again), p < 0.01; see Fig. 11). This reflected a stronger Stop-success (successful vs failed stops) effect on slow-alpha power relative to the go-RT effect (slow vs fast). In turn, this one-way interaction was qualified by a two-way interaction with Modality (F(1,47) = 5.3), p < 0.05), reflecting a significant two-way interaction for auditory (F(1,47) = 7.1, p < 0.05) but not for visual (p = 0.412). For cross-modal go-stops then, alpha is associated more strongly with proactive inhibition than with proactive slowing (for which the association was practically zero). For visual-visual go-stops, the association with proactive inhibition is much weaker, while that with proactive slowing, although even weaker, is slightly different from zero. In addition, no significant effects of topography were observed.

**Fig. 11.**
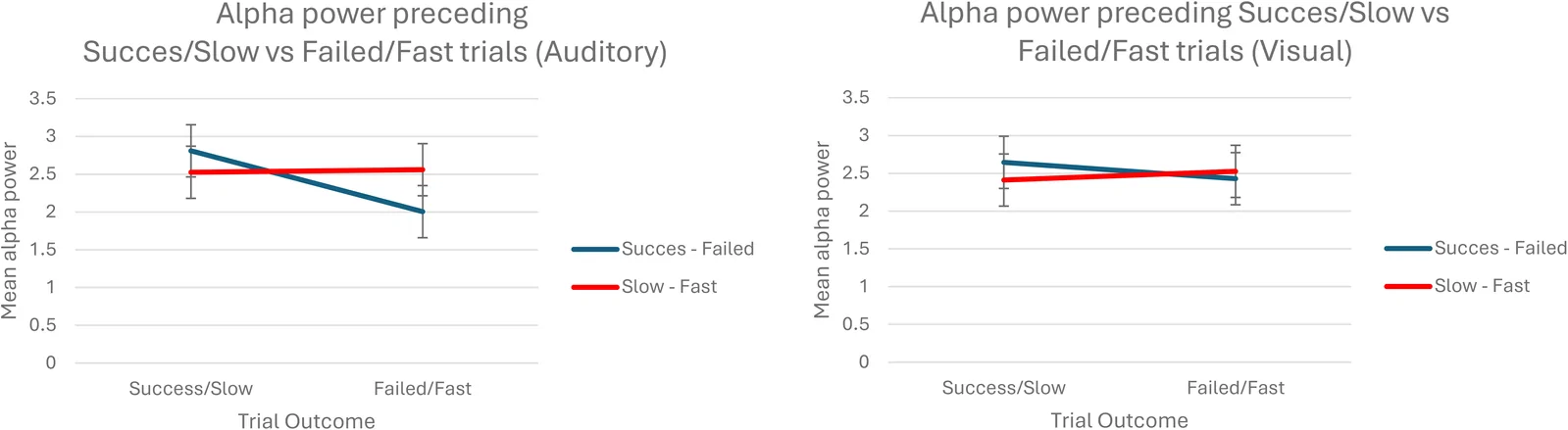
Slow-alpha power values as a function of Outcome (successful stop OR slow-RT/failed stop OR fast-RT; x axis) and Trial Selection (stop trials/go trials; separate lines), separated for the 2 modalities. Values are averages across all 28 electrodes.

## 4 Discussion

In this work, we investigated whether FM theta power preceding a stopping event was prospectively associated with stopping success. We demonstrate that in the context of an SST, FM theta is higher preceding successful stopping events than preceding failed stopping events. We also show that this is irrespective of the modality of the stop signal; we observe no difference between trials with visual versus auditory stop cues. Although the mean difference in theta power is small, it is consistent, and demonstrates the role of theta power in successful inhibition. We subsequently showed that FM theta power preceding all stopping events is associated with specifically the SSRT, with a higher theta being indicative of shorter SSRTs. However, a positive correlation between theta and go-RT was also found and is consistent with a proactive-slowing account. Moreover, a further analysis in which go trials were sorted according to go-RTs (slow versus fast) did in no way support a dissociation between stopping success and go-RT with respect to the association with FM theta power. Consistently, when comparing the identity of the previous trials between successful and failed stop trials, longer go-RTs preceding successful stop trials were observed. The only strong dissociation that was observed between SSRT and go-RT pertained to a lack of a correlation between the extent of theta being associated with stop success and that of theta being associated with go-RT. This suggests that whereas for one group of individuals, theta is associated more with proactive inhibition, for another it is associated more with proactive slowing.

These results seem to contradict those of Chang et al. (2017), who reported a specific association between a measure of stopping anticipation and pre-go theta power, while the latter was not associated with go RT. However, in the Chang study, this dissociation between stopping anticipation and response speed with respect to theta occurred despite a positive association between stopping anticipation and response speed within subjects. Together, this suggests that estimates of fluctuating subjective stop probability primarily affect the inhibition process itself through proactive control. In contrast, externally defined probabilities, as with stop-probability cues (Messel et al., 2021) or more global stop probability (Lansbergen et al., 2007), primarily induce proactive slowing. Indeed, a study by Dippel et al. (2017) suggests a potentially separate mechanism underlying increased theta power in the context of an externally defined inhibitory probability. These authors reported increased pupil diameter, which was linked to noradrenergic activity only when there was a high demand for inhibitory control.

The current results further indicate that when we sort our stop events based on difficulty (short vs long SOA), we find a larger mean difference in theta power between failed stop trials with a short SOA (easy) vs. successful stop trials with a long SOA (difficult), as compared with the main analyses including all trials. This finding indicates that participants showed higher FM theta preceding difficult stop trials with successful stops, compared with easy stop trials with failed stops. Note that trial numbers in the easy–difficult comparison were only half of those in the original comparison, which likely reduced the statistical power. Despite this reduced statistical power, we did find a clear difference.

As to the time course of the proactive theta power, it clearly peaked in the first half of the pre-go time period. Although stop-success effects were only significant in this same half period, this was not confirmed by a significant interaction between stop success and time. Apparently, participants differed in the exact timing of presenting larger theta responses preceding successful versus failed stops. Nevertheless, it can be concluded that a theta signal emerges somewhere after the closure of the preceding trial, and was fairly discrete in that it was not protracted across the whole of the pre-go epoch. Note that this held for both the association between theta and stop success, and for that between theta and go-RT.

This pattern begs the question of whether there are sequelae of this discrete theta signal that may reflect a potentiated inhibitory sensory-motor connection. Serendipitously, such a sequela was noted in the form of slow-alpha power, which was stronger in the second half of the epoch and for successful compared with failed stops. For slow alpha, the association with stop-success effect was much more pronounced than that with go-RT, the latter being virtually zero for cross-modal go-stop configurations. Given this pattern of results, the midfrontal slow-alpha power may reflect, perhaps indirectly, a potentiated inhibitory sensory-motor connection. The larger stop-success effect for auditory signals could be related to the shorter SSRTs for auditory than for visual. The exact mechanisms underlying these relations are beyond the scope of this work.

In an extensive topographic analysis, we did not find any sign of a contribution to theta or slow-alpha power from lateral–frontal sources. Although in a recent study, in a go/no-go context, Johari and Berger (2024) also reported pronounced theta-band activity in dorsal premotor areas, earlier studies did reveal combined contributions from medial and lateral sources (Asanowicz et al., 2022; Chang et al., 2017). Although the proactive component has been linked to right inferior frontal gyrus (rIFG, see Introduction), the present theta fronto-medially tinged topography fits much better a dorsal–medial pre-SMA intracranial source. This is especially evident when considering right-sided topographies presumed to reflect rIFG responses to the stop signal as reported by, for example, Schmajuk et al. (2006) and Kenemans et al. (2023). A preSMA-based proactive theta signal is somewhat surprising given the emphasis on rIFG-based proactivity in some theoretical discussions (e.g., Kenemans, 2015).

Another factor may be the latency of the stop-related proactive theta in Chang et al. (2017), which ranges from approximately (on average) 2,000 to 1,800 ms before the go stimulus, much more remote in time (relative to go) than the present -900 to -500 ms. This leaves open the possibility that, perhaps closer in time to the go stimulus, rIFG activity may play a proactive role as well. For example, a recent spatiotemporal combined EEG-fMRI decomposition analysis suggested that purportedly frontally midline-distributed theta preceding go stimuli was associated with rIFG activation, in a time window of approximately 1,300–900 ms pre-go stimulus (Messel et al., 2021). This is much closer in time than in the case of Chang et al. (2017), but still almost completely preceding the currently analyzed time window. Conspicuously, in this study, pre-go theta was elicited by cue stimuli indicating the probability of a stop signal. As argued above, this might affect more the extent of proactive slowing than of proactive inhibitory control. Several uncertainties should be noted, however, about the theta topography in the Messel et al. study that, firstly, did not show these topographies, and, secondly, may have produced distorted theta maps (as well as specific FCz values that were used in their analysis) due to average-referencing preceding Fourier/wavelet transforms (see, e.g., Schutte et al., 2017).

Taken together, based on our topographical analyses, a combined role of preSMA and rIFG in proactive inhibitory control and proactive slowing is conceivable and aligns with similar proposals from other authors (e.g., Aron & Poldrack, 2006). For instance, this could involve an initial preSMA activation in response to an internal or external trigger signaling an expectation of a go-stop combination. This activation might then relay signals to rIFG neurons, which, in turn, send potentiating signals to inhibitory sensory-motor connections (see Kenemans, 2015) as well as back to preSMA. It is also plausible that preSMA activation dominates the ensuing scalp distribution, thereby obscuring the relatively small contribution from rIFG—consistent with the relatively modest right-frontal activation observed prior to the pronounced dorsal–medial activation in response to successfully applied stop signals (Kenemans et al., 2023; Schmajuk et al., 2006). This would also be consistent with classic reports on impaired stopping (longer SSRTs) following rIFG lesions (Aron et al., 2003) and preSMA lesions (Floden & Stuss, 2006).

Interestingly, although a number of stimulant treatment-response predicting biomarkers have been proposed for ADHD, perhaps the most robust one is resting-state low frontal-alpha peak frequency (predicting clinical non-response; Arns, 2012). It could be speculated that dominant slow-alpha frequency during resting state is somehow associated with a low ability for poor slow-alpha synchronization in conditions demanding proactive impulse control, such as the current stop-signal conditions. Hence, further investigation of the role of FM theta and slow-alpha power in proactive inhibition could be specifically relevant for our understanding of altered response inhibition performance in clinical groups.

A final follow-up question that may be relevant in light of the current results concerns the role of tonic, resting-state theta power in modulating the theta response preceding an inhibitory event. Work by Pscherer et al. (2019) indicates that a low resting-state baseline theta power influences the active theta response to inhibitory demands in a version of the go/no-go task. Hence, baseline theta power may also play a role in determining proactive recruitment of theta power in a response inhibition context.

In conclusion, to the best of our knowledge, our study is the first to identify a positive relationship between FM theta power (as well as FM slow-alpha power) and subsequent stop success, highlighting its potential role in proactive response inhibition. At the level of group averages, theta, but not slow alpha, is equally strongly associated with proactive slowing, but the relative associations with inhibition and slowing, respectively, differ profoundly across individuals. Despite these individual differences, FM theta is associated with both proactive inhibition and proactive slowing. In contrast, FM slow alpha is more specifically associated with proactive inhibition, and also marked by a different time course relative to theta.

## Data Availability

Data and code are available upon request through the YODA Repository of the Faculty of Social Sciences, Utrecht University via the following doi: https://doi.org/10.24416/UU01-JI88TT.

## References

[IMAG.a.1390-b1] Asanowicz, D., Kotlewska, I., & Panek, B. (2022). Neural underpinnings of proactive and preemptive adjustments of action control. Journal of Cognitive Neuroscience, 34(9), 1590–1615. 10.1162/jocn_a_01884 35802602

[IMAG.a.1390-b2] Arns, M. (2012). EEG-based personalized medicine in ADHD: Individual alpha peak frequency as an endophenotype associated with nonresponse. Journal of Neurotherapy, 16(2), 123–141. 10.1080/10874208.2012.677664

[IMAG.a.1390-b3] Aron, A. R. (2011). From reactive to proactive and selective control: Developing a richer model for stopping inappropriate responses. Biological Psychiatry, 69(12), e55–e68. 10.1016/j.biopsych.2010.07.024 PMC3039712 20932513

[IMAG.a.1390-b4] Aron, A. R., Fletcher, P. C., Bullmore, E. T., Sahakian, B. J., & Robbins, T. W. (2003). Stop-signal inhibition disrupted by damage to right inferior frontal gyrus in humans. Nature Neuroscience, 6(2), 115–116. 10.1038/nn1003 12536210

[IMAG.a.1390-b5] Aron, A. R., & Poldrack, R. A. (2006). Cortical and subcortical contributions to stop signal response inhibition: Role of the subthalamic nucleus. Journal of Neuroscience, 26(9), 2424–2433. 10.1523/jneurosci.4682-05.2006 PMC6793670 16510720

[IMAG.a.1390-b6] Adelhöfer, N., & Beste, C. (2020). Pre-trial theta band activity in the ventromedial prefrontal cortex correlates with inhibition-related theta band activity in the right inferior frontal cortex. NeuroImage, 219, 117052. 10.1016/j.neuroimage.2020.117052 32540357

[IMAG.a.1390-b7] Adelhöfer, N., Bluschke, A., Roessner, V., & Beste, C. (2021). The dynamics of theta-related pro-active control and response inhibition processes in AD (H) D. NeuroImage: Clinical, 30, 102609. 10.1016/j.nicl.2021.102609 PMC7970141 33711621

[IMAG.a.1390-b8] Cai, W., Oldenkamp, C. L., & Aron, A. R. (2011). A proactive mechanism for selective suppression of response tendencies. Journal of Neuroscience, 31(16), 5965–5969. 10.1523/jneurosci.6292-10.2011 PMC3111595 21508221

[IMAG.a.1390-b9] Cavanagh, J. F., & Frank, M. J. (2014). Frontal theta as a mechanism for cognitive control. Trends in Cognitive Sciences, 18(8), 414–421. 10.1016/j.tics.2014.04.012 PMC4112145 24835663

[IMAG.a.1390-b10] Chang, A., Ide, J. S., Li, H. H., Chen, C. C., & Li, C. S. R. (2017). Proactive control: Neural oscillatory correlates of conflict anticipation and response slowing. Eneuro, 4(3), eneuro.0061-17.2017. 10.1523/eneuro.0061-17.2017 PMC5446487 28560315

[IMAG.a.1390-b11] Cooper, P. S., Karayanidis, F., McKewen, M., McLellan-Hall, S., Wong, A. S., Skippen, P., & Cavanagh, J. F. (2019). Frontal theta predicts specific cognitive control-induced behavioural changes beyond general reaction time slowing. NeuroImage, 189, 130–140. 10.1016/j.neuroimage.2019.01.022 PMC7664162 30639331

[IMAG.a.1390-b12] Cooper, P. S., Wong, A. S. W., Fulham, W. R., Thienel, R., Mansfield, E., Michie, P. T., & Karayanidis, F. (2015). Theta frontoparietal connectivity associated with proactive and reactive cognitive control processes. NeuroImage, 108, 354–363. 10.1016/j.neuroimage.2014.12.028 25528657

[IMAG.a.1390-b13] Cooper, P. S., Wong, A. S. W., McKewen, M., Michie, P. T., & Karayanidis, F. (2017). Frontoparietal theta oscillations during proactive control are associated with goal-updating and reduced behavioral variability. Biological Psychology, 129, 253–264. 10.1016/j.biopsycho.2017.09.008 28923361

[IMAG.a.1390-b14] De Jong, R., Coles, M. G., Logan, G. D., & Gratton, G. (1990). In search of the point of no return: The control of response processes. Journal of Experimental Psychology: Human Perception and Performance, 16(1), 164. 10.1037/0096-1523.16.1.164 2137517

[IMAG.a.1390-b15] Dippel, G., Mückschel, M., Ziemssen, T., & Beste, C. (2017). Demands on response inhibition processes determine modulations of theta band activity in superior frontal areas and correlations with pupillometry–Implications for the norepinephrine system during inhibitory control. NeuroImage, 157, 575–585. 10.1016/j.neuroimage.2017.06.037 28647483

[IMAG.a.1390-b16] Floden, D., & Stuss, D. T. (2006). Inhibitory control is slowed in patients with right superior medial frontal damage. Journal of Cognitive Neuroscience, 18(11), 1843–1849. 10.1162/jocn.2006.18.11.1843 17069475

[IMAG.a.1390-b17] Gratton, G., Coles, M. G., & Donchin, E. (1983). A new method for off-line removal of ocular artifact. Electroencephalography and Clinical Neurophysiology, 55(4), 468–484. 10.1016/0013-4694(83)90135-9 6187540

[IMAG.a.1390-b18] Johari, K., & Berger, J. I. (2024). Theta oscillations within right dorsolateral prefrontal cortex contribute differently to speech versus limb inhibition. Journal of Neuroscience Research, 102(2), e25298. 10.1002/jnr.25298/v2/response1 38361410

[IMAG.a.1390-b19] Kenemans, J. L. (2015). Specific proactive and generic reactive inhibition. Neuroscience & Biobehavioral Reviews, 56, 115–126. 10.1016/j.neubiorev.2015.06.011 26116545

[IMAG.a.1390-b20] Kenemans, J. L., Schutte, I., Van Bijnen, S., & Logemann, H. A. (2023). How salience enhances inhibitory control: An analysis of electro-cortical mechanisms. Biological Psychology, 177, 108505. 10.1016/j.biopsycho.2023.108505 36669616

[IMAG.a.1390-b21] Lansbergen, M. M., Schutter, D. J., & Kenemans, J. L. (2007). Subjective impulsivity and baseline EEG in relation to stopping performance. Brain Research, 1148, 161–169. 10.1016/j.brainres.2007.02.034 17362884

[IMAG.a.1390-b23] Majid, D. A., Cai, W., Corey-Bloom, J., & Aron, A. R. (2013). Proactive selective response suppression is implemented via the basal ganglia. Journal of Neuroscience, 33(33), 13259–13269. 10.1523/jneurosci.5651-12.2013 PMC3742918 23946385

[IMAG.a.1390-b24] Mazaheri, A., Coffey-Corina, S., Mangun, G. R., Bekker, E. M., Berry, A. S., & Corbett, B. A. (2010). Functional disconnection of frontal cortex and visual cortex in attention-deficit/hyperactivity disorder. Biological Psychiatry, 67(7), 617–623. 10.1016/j.biopsych.2009.11.022 20060100

[IMAG.a.1390-b25] Mazaheri, A., Fassbender, C., Coffey-Corina, S., Hartanto, T. A., Schweitzer, J. B., & Mangun, G. R. (2014). Differential oscillatory electroencephalogram between attention-deficit/hyperactivity disorder subtypes and typically developing adolescents. Biological Psychiatry, 76(5), 422–429. 10.1016/j.biopsych.2013.08.023 PMC3972379 24120092

[IMAG.a.1390-b26] Messel, M. S., Raud, L., Hoff, P. K., Stubberud, J., & Huster, R. J. (2021). Frontal-midline theta reflects different mechanisms associated with proactive and reactive control of inhibition. NeuroImage, 241, 118400. 10.1016/j.neuroimage.2021.118400 34311382

[IMAG.a.1390-b27] Pas, P., Pol, H. H., Raemaekers, M., & Vink, M. (2021). Self-regulation in the pre-adolescent brain. Developmental Cognitive Neuroscience, 51, 101012. 10.1016/j.dcn.2021.101012 PMC8450202 34530249

[IMAG.a.1390-b28] Pas, P., Van den Munkhof, H. E., Du Plessis, S., & Vink, M. (2017). Striatal activity during reactive inhibition is related to the expectation of stop-signals. Neuroscience, 361, 192–198. 10.1016/j.neuroscience.2017.08.037 28844007

[IMAG.a.1390-b29] Pscherer, C., Mückschel, M., Summerer, L., Bluschke, A., & Beste, C. (2019). On the relevance of EEG resting theta activity for the neurophysiological dynamics underlying motor inhibitory control. Human Brain Mapping, 40(14), 4253–4265. 10.1002/hbm.24699 PMC6865515 31219652

[IMAG.a.1390-b30] Schutte, I., Kenemans, J. L., & Schutter, D. J. (2017). Resting-state theta/beta EEG ratio is associated with reward-and punishment-related reversal learning. Cognitive, Affective, & Behavioral Neuroscience, 17, 754–763. 10.3758/s13415-017-0510-3 PMC5548847 28585018

[IMAG.a.1390-b31] Schutte, I., Schutter, D. J. L. G., & Kenemans, J. L. (2023). Individual differences in the effects of salience and reward on impulse control and action selection. International Journal of Psychophysiology, 193, 112239. 10.1016/j.ijpsycho.2023.08.012 37643661

[IMAG.a.1390-b32] Schmajuk, M., Liotti, M., Busse, L., & Woldorff, M. G. (2006). Electrophysiological activity underlying inhibitory control processes in normal adults. Neuropsychologia, 44(3), 384–395. 10.1016/j.neuropsychologia.2005.06.005 16095637

[IMAG.a.1390-b33] van Driel, J., Swart, J. C., Egner, T., Ridderinkhof, K. R., & Cohen, M. X. (2015). (No) time for control: Frontal theta dynamics reveal the cost of temporally guided conflict anticipation. Cognitive, Affective, & Behavioral Neuroscience, 15(4), 787–807. 10.3758/s13415-015-0367-2 PMC4639582 26111755

[IMAG.a.1390-b34] Verbruggen, F., Aron, A. R., Band, G. P., Beste, C., Bissett, P. G., Brockett, A. T., Brown, J. W., Chamberlain, S. R., Chambers, C. D., Colonius, H., Colzato, L. S., Corneil, B. D., Coxon, J. P., Dupuis, A., Eagle, D. M., Garavan, H., Greenhouse, I., Heathcote, A., Huster, R. J., … Boehler, C. N. (2019). A consensus guide to capturing the ability to inhibit actions and impulsive behaviors in the stop-signal task. eLife, 8, e46323. 10.7554/elife.46323.026 PMC6533084 31033438

[IMAG.a.1390-b35] Vink, M., Zandbelt, B. B., Gladwin, T., Hillegers, M., Hoogendam, J. M., van den Wildenberg, W. P., Du Plessis, S., & Kahn, R. S. (2014). Frontostriatal activity and connectivity increase during proactive inhibition across adolescence and early adulthood. Human Brain Mapping, 35(9), 4415–4427. 10.1002/hbm.22483 PMC6869143 24532023

[IMAG.a.1390-b36] Zandbelt, B. B., Bloemendaal, M., Neggers, S. F. W., Kahn, R. S., & Vink, M. (2013). Expectations and violations: Delineating the neural network of proactive inhibitory control. Human Brain Mapping, 34(9), 2015–2024. 10.1002/hbm.22047 PMC6869973 22359406

[IMAG.a.1390-b37] Zavala, B., Jang, A., Trotta, M., Lungu, C. I., Brown, P., & Zaghloul, K. A. (2018). Cognitive control involves theta power within trials and beta power across trials in the prefrontal-subthalamic network. Brain, 141(12), 3361–3376. 10.1093/brain/awy266 PMC6272116 30358821

